# Comparing Lung CT in COVID-19 Pneumonia and Acute Heart Failure: An Imaging Conundrum

**DOI:** 10.7759/cureus.15120

**Published:** 2021-05-19

**Authors:** Leonardo P Suciadi, Yonathan William, Patricia Jorizal, Vera N Tarigan, Andreas H Santoso, Joshua Henrina, Firman Tedjasukmana, Nathania M Kristanti

**Affiliations:** 1 Cardiologist, Siloam Hospital Kebon Jeruk/Siloam Heart Institute, Jakarta, IDN; 2 Radiology, Siloam Hospital, Jakarta, IDN; 3 Family Medicine, Siloam Hospital, Jakarta, IDN; 4 Cardiology, Siloam Hospital, Jakarta, IDN

**Keywords:** lung ct, imaging, covid-19, pneumonia, heart failure

## Abstract

Background

Chest computed tomography (CT) provides an effective modality to evaluate patients with suspected coronavirus disease 2019 (COVID-19). However, overlapping imaging findings with cardiogenic pulmonary edema is not uncommon. Reports comparing the chest CT features of these diseases have not been elaborated. Thus, we aimed to show the difference between the low-dose lung CT findings of COVID-19 pneumonia and comparing them to those with acute heart failure (HF).

Methods

This retrospective analysis enrolled hospitalized patients with COVID-19 (n=10) and acute heart failure (n=9) that exclusively underwent low-dose chest CT scans within 24 hours of admission. Clinical and lung CT characteristics were collected and analyzed.

Results

The appearance of ground-glass-opacities (GGOs) has been recorded in all individuals in the HF and COVID-19 groups. There was no significant statistical difference between the two groups for rounded morphology, consolidation, crazy paving pattern, lesion distribution, and parenchymal band (P> 0.05). However, diffuse lesions were more frequent in HF cases (55.6% vs. 0%) than in COVID-19 pneumonia, which had a predominantly multifocal pattern. Notably, CT images in HF patients were more likely to have signs of interstitial tissue thickening, such as the interlobular septums, fissures, and peribronchovascular interstitium (55.6% vs 0%, 88.9% vs 20% and 44.4% vs 0%, respectively), as well as cardiomegaly (77.8% vs 0%), increased artery to bronchus ratio (55.6% vs 0%), and pleural effusions (77.8% vs 0%).

Conclusions

Major overlaps of lung CT imaging features existed between COVID-19 pneumonia and acute HF cases. However, signs of fluid redistribution are clues that favor HF over COVID-19 pneumonia.

## Introduction

Coronavirus disease 2019 (COVID-19) is a disease caused by a new strain of coronavirus that was first discovered at the end of December 2019 at Wuhan, Hubei province, China [1]. Later, this ribonucleic acid (RNA)-enveloped virus, identified as severe acute respiratory syndrome coronavirus-2 (SARS-CoV-2) spread rapidly around the world and caused a global pandemic [2]. Pneumonia, as stated in its name, is a major clinical manifestation in COVID-19. However, multiorgan dysfunctions have been reported at the later stages of the disease. Additionally, the spectrum of clinical manifestations is vast, ranging from asymptomatic/mild disturbances to critical status [3-5]. Thus, early detection and isolation are of paramount importance in tackling the spreading of this contagious illness to the community. Lung computed tomography (CT) imaging provides a useful tool to evaluate this viral pneumonia, even in the early stages. Several reports from initial cases in China and elsewhere have shown imaging characteristics on lung CT that can be used to identify COVID-19 pneumonia in clinical practice [6].

On the other hand, heart failure (HF) is one of the most frequent causes of hospitalization worldwide, especially among the elderly [7-8]. Pulmonary edema demonstrated in acute heart failure might appear as several abnormalities in chest X-ray (CXR) and lung CT, which is often challenging to differentiate with other lung diseases, including viral pneumonia [9]. These facts could cause uncertainty for frontline clinicians in making decisions regarding triage and treatment, as they have significantly different downstream management, particularly during this pandemic where reverse transcriptase-polymerase chain reaction (RT-PCR) may have a long turnaround time. Regarding this challenging issue, we collected several imaging data on lung CT of COVID-19 pneumonia and acute heart failure patients to further analyze whether these imaging features may help in differentiating these disease entities.

## Materials and methods

Subjects and study design

We enrolled hospitalized patients with a diagnosis of COVID-19 pneumonia or acute heart failure who underwent a low-dose chest computed tomography (CT) scan at the radiology department in Siloam Hospitals Kebon Jeruk, West Jakarta. It is important to note that our hospital is not a referral facility for managing COVID-19 cases. However, as it is located in a populous region with one of the highest numbers of cases in Jakarta, we encountered and managed numerous COVID-19 patients before they were transferred to referral hospitals.

Exclusively, subjects who underwent low-dose lung CT scans within the first 24-hour admission were included to reduce bias from early treatment administered, which might potentially alter the imaging findings, particularly in the heart failure group. Data were collected retrospectively from March 15 to April 28, 2020. Clinical profiles, laboratory results, and lung CT scans were recorded from medical records as well as the Pictures Archiving and Storage System (PACS). Investigators consisting of three cardiologists and three radiologists assessed the available data independently.

The diagnosis of COVID-19 was confirmed by real-time polymerase chain reaction (RT-PCR) (several kits and reagents) from a nasopharyngeal swab. The diagnosis of COVID-19 was established by at least one positive result for each test. Meanwhile, the diagnosis of acute heart failure was built regarding clinical findings, biomarkers (NTproBNP), and echocardiography examinations. The criteria for identifying HF were based on the Acute and Chronic Heart Failure Guidelines of the European Society of Cardiology (ESC) 2016 [10]. Candidates with less than an 18-year-old age or undergoing other protocols of lung CT scanning (e.g., standard dose high-resolution CT) were excluded. Furthermore, HF patients with any positive results of diagnostic tests for COVID-19 or other pneumonia infections were also ruled out.

The Hospital Ethical Review Board has reviewed and approved the study and waived the requirement of patients’ informed consent in accordance with the Council for International Organizations of Medical Sciences (CIOMS) guideline.

Imaging protocols

All patients underwent low-dose chest CT by using a Somatom Drive, dual-source 2x 128-slices 0.6 mm detector scanner (Siemens Healthineers, Forchheim, Germany). Two low-dose protocols were used, depending on the subject’s body weight: (1) For subjects less than 80 kg: Sn100 kilovoltage peak (kVp), 70 mAs, pitch 1.9, gantry rotation time 0.28 s, and (2) for subjects over 80 kg: Sn140 kVp, 40 mAs, pitch 1.55, gantry rotation time 0.28 s. Protocol (1) resulted in a CT dose index volume (CTDIvol) of 0.43 mGy while protocol (2) resulted in a CTDIvol of 1.14 mGy. The effective radiation dose was calculated by multiplying the dose-length product (DLP) by 0.014 mSv/mGy.cm (constant k-Value for chest CT scan). Images were reconstructed at 1.0 mm slice thickness and 1.0 mm increment using br59 sharp kernel, lung window (width 1200; level -600), ADvanced Modeled Iterative REconstruction (ADMIRE) strength five and bf37 smooth kernel, mediastinum window (width 350; level 50), ADMIRE strength 3. All reconstructions were performed with a field of vision (FOV) of 500 mm and a matrix size of 512x512.

Blinded image interpretation

All CT images were independently reviewed by three radiologists (YW, VN, and PJ). Radiological findings were noted as the presence of ground-glass-opacities (GGOs), crazy paving pattern, consolidation, parenchymal band, interlobular septal thickening, peribronchovascular interstitium thickening, cardiomegaly, enlarged main pulmonary artery, increased artery to bronchus ratio, venous redistribution, pleural effusion, pulmonary vein enlargement, superior vena cava enlargement, inferior vena cava enlargement, and azygos vein enlargement. The detailed definitions for all radiological endpoints have been previously described [11-12]. Further characteristics of ground-glass-opacities, crazy paving, and consolidation were elaborated such as rounded morphology, peripheral distribution, central distribution, AP-gradient distribution, diffuse pattern, focal/multifocal pattern, bilateral lung involvement, and multi-lobar involvement (including presence in each lobe).

Statistical analysis

All statistical procedures were perform using the Statistical Package for the Social Sciences 22.0 for Windows (IBM Corp., Armonk, NY). Means for continuous variables were compared using the independent group t-test when the data were normally distributed; otherwise, the Mann-Whitney test was used. The Shapiro-Wilk test was used to test the normality of data distribution. Proportions for categorical variables were compared using the Fisher exact test.

## Results

Clinical profile

We collected data from 10 patients with COVID-19 pneumonia and nine subjects with acute HF. Patients with HF were significantly older, with a mean age of 64.7 years old as compared to patients with COVID-19 pneumonia (mean age: 48.2 years). Slightly more than half of HF patients were hospitalized in the intensive care unit (ICU), whereas intensive care was not necessary for all subjects with COVID-19 (Table 1). Nevertheless, endotracheal intubation and mechanical ventilator support were not indicated for all of our subjects.

**Table 1:** Demographic and clinical characteristics of patients with heart failure and COVID-19 Mean ± SD, n (%), ICU = Intensive Care Unit, SpO2 = oxygen saturation, Hb = Hemoglobin, N/L ratio = Neutrophil/Lymphocyte ratio, GIT = Gastrointestinal tract, ESR = Erythrocyte sedimentation rate, eGFR = estimated glomerular filtration rate, CRP = C-reactive protein, NT-proBNP = N-terminal pro B-type natriuretic peptide, HF = Heart failure, CAD = Coronary artery disease, HCM = Hypertrophic cardiomyopathy ^a^Mann-Whitney test. Other P-values were obtained by independent t-test

	Heart Failure (n = 9)	COVID-19 (n =10)	P-Value
Age, years	64.7 ± 12	48.2 ± 10	0.005
Male	7 (77.8 %)	5 (50 %)	0.350
ICU stay	5 (55.6)	0 (0)	0.011
Intubation	0 (0)	0 (0)	-
Fever (>37.5^0^ C)	2 (22.2)	7 (70)	0.070
Dyspnea	5 (55.6)	1 (10)	0.057
Excessive fatigue	0 (0)	1 (10)	1.000
Cough	0 (0)	2 (20)	0.474
GIT symptoms	0 (0)	1 (10)	1.000
SpO2(%)	91.9 ± 6	96.5 ± 5	0.049^a^
Hb	11.3 ± 3	14.1 ± 1	0.026
Leucocyte	11055.5 ± 4090	6070.0 ± 2530	0.005
Neutrophil	73.9 ± 22	67.7 ± 12	0.130^a^
Lymphocyte	15.2 ± 9	25.7 ± 10	0.033
N/L ratio	7.3 ± 5	3.4 ± 2	0.072^a^
ESR	24.9 ± 28	36.6 ± 25	0.165^a^
Platelet	214333.3 ± 136010	220700 ± 97656	0.907
Creatinine	4.2 ± 5.8	0.9 ± 0.2	0.007^a^
eGFR	40.6 ± 27.3	87.9 ± 16.6	0.001
CRP	73.2 ± 62.5	94.6 ± 107.6	0.958^a^
NT-ProBNP	3587.5 ± 4870.9	-	-
HF aetiology:			
CAD	3 (33.3)		
Hypertension	3 (33.3)		
HCM	1 (11.1)		
Valvular	1 (11.1)		

Regarding the symptoms, fever above 37.5 °C was the most frequent complaint in the COVID-19 group (70% vs. 22.2%). Meanwhile, more HF patients experienced significant shortness of breath than in the COVID-19 group (55.6% vs. 10%). Other symptoms, including fatigue, gastrointestinal problems, and cough, were often found in patients with COVID-19 pneumonia. Moreover, HF patients tended to have lower initial O2 saturation, more anemia, higher leukocyte and neutrophil/lymphocyte ratio, and lower estimated GFR compared to COVID-19 counterparts. Besides, there were no statistical differences between groups with respect to the level of inflammation markers, including erythrocyte sedimentation rate (ESR) and C-reactive protein (CRP).

The majority of HF subjects were admitted as an acute decompensated episode on their chronic congestion, irrespective of left ventricular ejection fraction (LVEF). Mean LVEF in the HF group was 39.7 ± 19 %, whereas the mean N-terminal pro B-type natriuretic peptide (NT-proBNP) levels were 3587.5 ± 4870.9 ng/L. Coronary artery diseases (CAD) and hypertension contributed to one-third of HF etiology for each. Other HF cases were caused by a valvular abnormality (mitral valve prolapse with severe mitral regurgitation), burnout phase of hypertrophic cardiomyopathy, and high-output state (severe anemia).

Imaging features

Interestingly, GGO appearance has been recorded in all subjects in the HF and COVID-19 groups (Table 2). Additionally, there was no significant statistical difference between the two groups for rounded morphology, consolidation, crazy paving pattern, as well as a parenchymal band (P> 0.05). On the contrary, HF patients were more likely to have a thickening of the interlobular septum (55.6% vs. 0%), fissures (88.9% vs. 20%), and peribronchovascular interstitium (44.4% vs. 0%) on low-dose lung CT as compared to subjects with COVID-19 pneumonia.

**Table 2:** Comparison of imaging features in heart failure and COVID-19 pneumonia Values are n (%); ^a^Fisher exact test

	Heart Failure (n = 9)	COVID-19 (n = 10)		P-value^a^
Ground-glass opacities	9 (100 %)		10 (100 %)	-
Crazy paving pattern	3 (33.3)		2 (20)	0.628
Consolidation	7 (77.8)		6 (60)	0.628
Parenchymal band	5 (55.6)		5 (50)	1.000
Rounded morphology	3 (33.3)		1 (10)	0.303
Interlobular septal thickening	5 (55.6)		0 (0)	0.011
Fissural thickening	8 (88.9)		2 (20)	0.005
Peribronchovascular thickening	4 (44.4)		0 (0)	0.033
Peripheral distribution	7 (77.8)		10 (100)	0.211
Central distribution	6 (66.7)		2 (20)	0.070
Anteroposterior gradient distribution	1 (11.1)		0 (0)	0.474
Diffuse pattern	5 (55.6)		0 (0)	0.011
Focal pattern	4 (44.4)		10 (100)	0.011
Bilateral lung involvement	8 (88.9)		10 (100)	0.474
Multilobar involvement	8 (88.9)		10 (100)	0.474
Right upper lobe involvement	8 (88.9)		7 (70)	0.582
Right middle lobe involvement	7 (77.8)		6 (60)	0.628
Right inferior lobe involvement	8 (88.9)		9 (90)	1.000
Left upper lobe involvement	8 (88.9)		9 (90)	1.000
Left lower lobe involvement	9 (100)		9 (90)	1.000
Cardiomegaly	7 (77.8)		0 (0)	0.001
Enlarged main pulmonary artery	2 (22.2)		0 (0)	0.211
Increased artery to bronchus ratio	5 (55.6)		0 (0)	0.011
Venous redistribution/ cephalization	3 (33.3)		0 (0)	0.087
Pleural effusion	7 (77.8)		0 (0)	0.001
Pulmonary vein enlargement	3 (33.3)		0 (0)	0.087
Superior vena cava (SVC) enlargement	1 (11.1)		0 (0)	0.474
Inferior vena cava (IVC) enlargement	1 (11.1)		0 (0)	0.474
Azygos vein enlargement	1 (11.1)		0 (0)	0.474

Cardiogenic lung edema tended to have a more central distribution (66.7% vs. 20%) while COVID-19 pneumonia showed a more peripheral distribution (100% vs. 77.8%) although the difference was not statistically significant. Furthermore, diffuse lesions were more frequent in HF cases than in viral pneumonia (55.6% vs. 0%), whereas the focal pattern was more likely for COVID-19 pneumonia (100% vs. 44.4%). Bilateral lung and multilobar involvement were common for both conditions, and there was no predilection for a particular lobe affected by each disease.

It is noteworthy that findings of cardiomegaly, an increased artery to bronchus ratio, and pleural effusion were remarkably indicative for HF (77.8% vs. 0%, 55.6% vs. 0%, and 77.8% vs. 0%, respectively), as none of such signs were detected in our subjects with COVID-19 pneumonia. Central vein enlargement and venous cephalization were rarely viewed for both conditions. Perhaps, the significant overlap between the normal and abnormal range of diameter, as well as the variable diameter during inspiration, limited the usefulness of low-dose CT in evaluating chest veins.

## Discussion

Microbiologic testing of the respiratory tract swab with RT-PCR is still the mainstay for a definitive diagnosis of SARS-CoV-2 infection. However, such a procedure has been reported to offer relatively low sensitivity (60%-90%) in detecting the infection, especially at the initial stage [13]. The positive rates are varied by sample or specimen tested, and bronchoalveolar lavage (BAL) fluid has the highest sensitivity of 93% [14]. In our region, there was a shortage of RT-PCR testing availability in the early pandemic. The RT-PCR turnaround time ranged between one and 14 days, severely limiting their usefulness in clinical settings. Decisions were mainly guided by clinical and imaging findings while RT-PCR only served as a confirmatory diagnosis. Therefore, to optimize diagnostic speed and accuracy in patients suspected of COVID-19, low-dose lung CT was used in most cases at our center, instead of chest X-ray (CXR).

Previous reports have revealed that lung CT scanning is excellent to detect COVID-19 pneumonia since the early stage, even in patients with asymptomatic or mild symptoms. The sensitivity of lung CT imaging is beyond conventional CXR in recognizing COVID-19 in an earlier phase and in mild abnormalities [15]. Notwithstanding, CT scanning is relatively costly, restricted in availability, involves ionizing radiation, and cumbersome to use in high-volume demands in such contagious disease. The low-dose lung CT protocol is commonly used for lung cancer screening to reduce the radiation dose, mostly 0.2-0.8 mSv (depending on the individual patient) as compared to the usual 2.0 to 6.0 mSv using standard-dose chest CT and about <0.1 mSv for frontal CXR. Using low-dose lung CT may also reduce cost, as we charged it at a lower expense than high-resolution CT imaging (low-dose lung CT is charged at 80 USD while standard-chest CT is charged at 190 USD).

**Figure 1:**
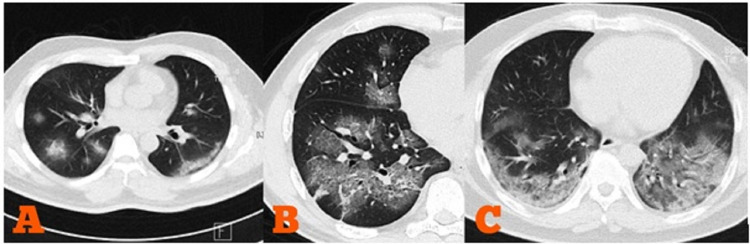
Characteristic findings of low-dose chest CT imaging in COVID-19 pneumonia found in our patients A. Multifocal and bilateral rounded GGO with predominant peripheral distribution; B. Crazy paving pattern; C. Bilateral mixed GGO and consolidation with posterobasal predominance GGO = ground-glass-opacity

According to previous reports, the characteristic findings of lung CT scans of individuals with COVID-19 are multifocal and bilateral GGOs with or without consolidation or crazy paving pattern, mostly distributed in the peripheral fields of the lungs [16-17]. A reverse halo sign or other findings of organizing pneumonia could also be viewed. The most typical of these features are rounded and patchy patterns (Figure 1) [18]. Bai et al. reported that GGOs and peripheral distribution accounted for 91% and 80% cases, respectively [19]. On the contrary, discrete small nodules, isolated lobar or segmental consolidation without GGO, lymphadenopathy, cavitation, and pleural effusion are less common findings on lung CT imaging of COVID-19 patients.

Lung CT features can vary over time as the disease progresses so that they can also indicate disease evolution [20]. In the early phase, multiple, small, patchy GGOs and interstitial changes appeared in subpleural locations. However, more than half of the patients had normal lung CT findings within the first 48 hours after the onset of symptoms [21]. In the progressive phase, the lesions enlarge and evolve into multiple GGOs, crazy paving, and consolidations in the lung. Diffuse consolidations showing as a white lung with a persistent air bronchogram can be found in severe cases. These opacities are eventually absorbed in the recovery period, but subpleural fibrosis may remain [22-23].

GGO is a nonspecific feature in the lungs on CT imaging, referring to an area with increased attenuation, commonly as haziness, with preserved bronchial and vascular markings [24]. It can be formed by various mechanisms, including a partial filling of alveoli by any fluid, interstitial thickening, or partial collapse of alveoli. Meanwhile, a crazy-paving pattern refers to the superimposition of irregular linear structures on a background of GGO. The linear morphology of this pattern is contributed by interlobular or intralobular septal thickening and interstitial arrangement [25]. This feature is also nonspecific and commonly found on high-resolution lung CT in many diseases. Hence, both aforementioned patterns are not pathognomonic for COVID-19 pneumonia, as those can be seen on numerous pathologies, including cardiogenic pulmonary edema, all causes of organizing pneumonia, and all causes of diffuse alveolar damage, including pneumonitis, sarcoidosis, and pulmonary hemorrhage. 

As mentioned before, similar abnormalities on lung CT can be viewed in a patient with congestive heart failure. GGO, crazy paving pattern, consolidation, perivascular haziness, and septal and fissural thickening are frequently recognized in cardiogenic lung edema, along with cardiomegaly and pleural effusions. However, the incidence and predominance of these features are varied among HF patients [9]. Central distribution of opacities is more typical for heart failure, but patchy GGO is not uncommon in some patients. Because lung opacities in HF is caused by hydrostatic pulmonary edema, the gravitational effect plays a role in the distribution of this feature to dependent areas of the lungs. The perihilar or bat-wing appearance of edema distribution is more often found in pulmonary edema, in which rapid accumulation of fluid occurs in the lungs. High-resolution CT imaging may sometimes add further information about the engorgement of central veins, such as pulmonary veins, Azygos veins, inferior vena cava (IVC), and superior vena cava (SVC), indicating fluid overload in HF [23].

This study showed that distinguishing COVID-19 pneumonia and heart failure based on low-dose lung CT imaging can be challenging because of some overlapping features in imaging patterns between these diseases. GGO, crazy paving pattern, and consolidation were all common in COVID-19 pneumonia and HF as well. Therefore, these dramatic patterns should not be considered as specific signs for COVID-19 pneumonia. Furthermore, to put it into a more complex situation, our report also revealed that certain lesion distribution and morphology could not be used easily to differentiate between the two. Our data showed that lesions caused by cardiogenic lung edema had a tendency to be centralized in distribution. However, it was not statistically significant (66.7% vs. 20%, P-value 0.07). Perhaps, the diffuse distribution toward peripheral sites seen in this study was related to the majority of these HF patients who had long-term HF status with chronic congestion, not acute de novo cases with flash pulmonary edema.

**Figure 2:**
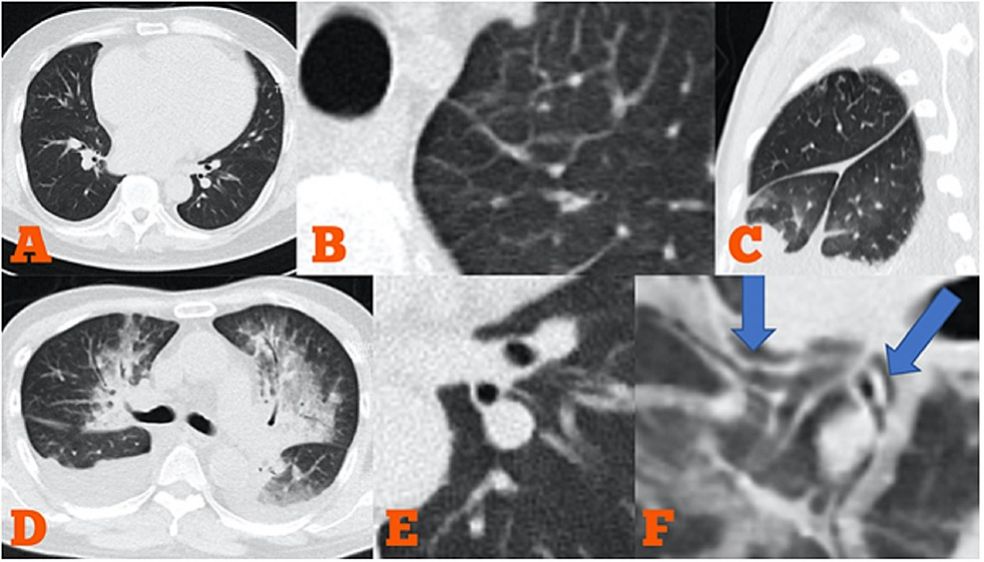
Typical imaging features in congestive heart failure A. Cardiomegaly; B. Interlobular septal thickening; C. Fissural thickening; D. Mixed GGO and consolidation with a central distribution. Significant bilateral pleural effusion also existed; E. Increased artery-bronchus ratio; F. Peribronchovascular interstitial thickening at the lower lobe bronchus GGO:  ground-glass opacity

Importantly, identifying several signs indicating fluid redistribution or pulmonary edema as the result of heart failure might help to discern it from COVID-19 pneumonia. Those signs included interlobular septal thickening, fissural thickening, peribronchovascular thickening, increased artery to bronchus ratio, pleural effusion (especially bilateral), and cardiac enlargement in more advanced HF (Figure 2). Lung lesions in HF patients were more likely to be diffuse compared to COVID-19 pneumonia in which focal pattern may be often viewed. Enlargement of the central veins system might be identified on chest CT, however, a significant overlap between the normal and abnormal range of diameter, as well as variable diameter during inspiration, has limited the usefulness of CT in evaluating chest veins.

These overlap findings on low-dose lung CT between COVID-19 pneumonia and HF could potentially raise uncertainty in clinical decisions amidst this pandemic, mainly if the presenting symptoms of COVID-19 are vague and there is uncertain history contact with suspects. As a consequence, HF patients with the aforementioned lung CT features could be suspected as COVID-19 pneumonia at initial presentation resulting in delayed management for HF that might worsen the prognosis of these fragile patients. In another scenario, COVID-19 infection might become precipitation for acute decompensated HF. If there is no clinical suspicion in this situation, the diagnosis of COVID-19 pneumonia on top of acute HF could be missed, particularly if additional tests other than lung CT imaging are not performed to screen the infection. It has to be noted that RT-PCR is still the reference test for diagnosing COVID-19. However, the availability of RT-PCR testing was limited in our region, particularly in the early pandemic days. In such a situation, a CT scan may assist in patient triage. Despite its limited specificity, CT imaging has been recognized to be highly sensitive for patients with an indication for hospitalization, and there are still CT features that may favor the presence of COVID-19 and may further guide the clinician to make a better clinical decision regarding downstream management.

In general, there are no typical symptoms for COVID-19, and negligence is common in patients with mild symptoms or asymptomatic. The majority of subjects with COVID-19 pneumonia in this study had a fever above 37.5 °C, whereas HF patients were more commonly admitted with prominent dyspnea. Notably, low-grade fever might also occur in HF, especially in severe and advanced conditions. A slight increase in body temperature is possibly related to elevated metabolic rate, over-sympathetic regulation, and chronic inflammation status in the HF setting [24-25]. Diagnosis COVID-19 is more straightforward in young patients presented with obvious symptoms of respiratory tracts and significant fever, especially if contact history is recognized because the prevalence of HF in age younger than 55 years old is relatively rare [8].

There are several limitations to this study. First, this is a retrospective and cross-sectional design, thus clinical and CT imaging follow-up after receiving therapy has not been conducted. Second, the number of participated subjects is limited, as our center is not designated as a referral hospital. This situation can cause a substantial problem regarding the generalizability of these research findings. Further prospective research with an ample number of subjects are needed. Third, subjects with COVID-19 had presumably non-severe symptoms because no respiratory failure cases that required ventilation support were noted. However, it was comparable with subjects in the HF group whom there were no one ended in intubation. Finally, there was a significant age discrepancy between the two groups in which HF subjects were much older. A pre-existing chronic pulmonary disease might be present in several subjects with HF regarding aging or previous history. This comorbid was potentially affect lung CT findings in this HF group.

## Conclusions

Chest CT is useful in the early detection and evaluation of COVID-19 pneumonia during the pandemic. However, distinguishing COVID-19 pneumonia and HF may not always be straightforward due to CT features overlap. Features of GGO, crazy paving pattern, and consolidation, with or without certain lesion distribution or morphology, were all common in both diseases. Nevertheless, there are still some CT characteristics that may favor HF over viral pneumonia. Identifying signs of fluid redistribution as the result of heart failure might offer a clue to differentiate it from COVID-19 pneumonia. Thickening of the interlobular septa, fissures, and peribronchovascular markings, increased artery to bronchus ratio, cardiomegaly, and pleural effusion are more often encountered in HF patients.
